# ChemFOnt: the chemical functional ontology resource

**DOI:** 10.1093/nar/gkac919

**Published:** 2022-10-28

**Authors:** David S Wishart, Sagan Girod, Harrison Peters, Eponine Oler, Juan Jovel, Zachary Budinski, Ralph Milford, Vicki W Lui, Zinat Sayeeda, Robert Mah, William Wei, Hasan Badran, Elvis Lo, Mai Yamamoto, Yannick Djoumbou-Feunang, Naama Karu, Vasuk Gautam

**Affiliations:** Department of Biological Sciences, University of Alberta, Edmonton, AB T6G 2E9, Canada; Department of Computing Science, University of Alberta, Edmonton, AB T6G 2E8, Canada; Department of Laboratory Medicine and Pathology, University of Alberta, Edmonton, AB T6G 2B7, Canada; Faculty of Pharmacy and Pharmaceutical Sciences, University of Alberta, Edmonton, AB T6G 2H7, Canada; Department of Biological Sciences, University of Alberta, Edmonton, AB T6G 2E9, Canada; Department of Biological Sciences, University of Alberta, Edmonton, AB T6G 2E9, Canada; Department of Biological Sciences, University of Alberta, Edmonton, AB T6G 2E9, Canada; Department of Biological Sciences, University of Alberta, Edmonton, AB T6G 2E9, Canada; Department of Biological Sciences, University of Alberta, Edmonton, AB T6G 2E9, Canada; Department of Computing Science, University of Alberta, Edmonton, AB T6G 2E8, Canada; Department of Biological Sciences, University of Alberta, Edmonton, AB T6G 2E9, Canada; Department of Biological Sciences, University of Alberta, Edmonton, AB T6G 2E9, Canada; Department of Biological Sciences, University of Alberta, Edmonton, AB T6G 2E9, Canada; Department of Biological Sciences, University of Alberta, Edmonton, AB T6G 2E9, Canada; Department of Biological Sciences, University of Alberta, Edmonton, AB T6G 2E9, Canada; Department of Biological Sciences, University of Alberta, Edmonton, AB T6G 2E9, Canada; Molecular You Corporation, 788 Beatty St., Suite 307, Vancouver, BC V6B 2M1, Canada; Corteva Agriscience, Indianapolis, IN 46268, USA; Leiden Academic Centre for Drug Research, Leiden University, Leiden, 2333 CC, The Netherlands; Department of Biological Sciences, University of Alberta, Edmonton, AB T6G 2E9, Canada

## Abstract

The **Chem**ical **F**unctional **Ont**ology (ChemFOnt), located at https://www.chemfont.ca, is a hierarchical, OWL-compatible ontology describing the functions and actions of >341 000 biologically important chemicals. These include primary metabolites, secondary metabolites, natural products, food chemicals, synthetic food additives, drugs, herbicides, pesticides and environmental chemicals. ChemFOnt is a FAIR-compliant resource intended to bring the same rigor, standardization and formal structure to the terms and terminology used in biochemistry, food chemistry and environmental chemistry as the gene ontology (GO) has brought to molecular biology. ChemFOnt is available as both a freely accessible, web-enabled database and a downloadable Web Ontology Language (OWL) file. Users may download and deploy ChemFOnt within their own chemical databases or integrate ChemFOnt into their own analytical software to generate machine readable relationships that can be used to make new inferences, enrich their omics data sets or make new, non-obvious connections between chemicals and their direct or indirect effects. The web version of the ChemFOnt database has been designed to be easy to search, browse and navigate. Currently ChemFOnt contains data on 341 627 chemicals, including 515 332 terms or definitions. The functional hierarchy for ChemFOnt consists of four functional ‘aspects’, 12 functional super-categories and a total of 173 705 functional terms. In addition, each of the chemicals are classified into 4825 structure-based chemical classes. ChemFOnt currently contains 3.9 million protein-chemical relationships and ∼10.3 million chemical-functional relationships. The long-term goal for ChemFOnt is for it to be adopted by databases and software tools used by the general chemistry community as well as the metabolomics, exposomics, metagenomics, genomics and proteomics communities.

## INTRODUCTION

Chemicals are essential to life. They play key roles in signalling, communication, energy generation, metabolism, catabolism and anabolism—including the assembly and synthesis of macromolecules (DNR, RNA, proteins, lipids), cells and tissues. In many respects, chemicals function as the cell's bricks and mortar while at the same time providing the fuel to sustain those ‘bricklaying’ functions. Chemicals can also serve as drugs, poisons, nutrients, biomarkers, waste products, flavorants, odorants, colorants or dyes, repellants, catalysts, buffers, stabilizers, antioxidants, flocculants and a host of other roles. Understanding the roles and functions of chemicals is vital to understanding many areas of chemistry, biochemistry, molecular biology and biology. Likewise, understanding the origins of chemicals in foods, cosmetics, drugs, the environment and in our own bodies and how they affect both ourselves and other living organisms is of considerable interest to anyone doing life science research. Unfortunately, this kind of information, especially that on the functional effects, processes, roles or origins (disposition) of chemicals, is often diffusely scattered as snippets of text, lists or tables contained in thousands of books, journals, online databases that span decades or even centuries of publication. The fact that all this functional information still resides in books and journals with no formal descriptions, limited consensus definitions and no machine-readable structure means that a significant amount of biologically important chemistry is inconsistently described, improperly annotated, ill-defined—and unfortunately, inaccessible.

One approach to correct this kind of information deficit is to develop an electronic ontology. An ontology comprises a series of named entities, their formal descriptions or definitions, their relationships to each other and a series of hierarchical categories that group those entities according to various criteria. Perhaps the best-known ontology in biology is the gene ontology (GO), which was developed by Michael Ashburner *et al.* (1). GO was developed to unify the description of gene and protein attributes to help create a more comprehensive, computational model of biological systems (1,2). More specifically, GO did three important things: (i) it developed a controlled vocabulary and a well-designed hierarchy of gene and protein attributes; (ii) it annotated millions of genes and proteins using that controlled vocabulary and (iii) it provided software tools to assimilate and disseminate that annotation data for easy access to all. GO also introduced three broad and widely used terms to describe genes and/or proteins covering their molecular function, cellular component and biological process. GO is widely used to help in the analysis and interpretation of large-scale genomics, transcriptomics and proteomics experiments (3,4). Currently, GO has 43 558 defined terms, which have been used to create 7.48 million annotations for 1.48 million genes/proteins from 5200 species (http://geneontology.org/).

Notably absent from the GO effort was the inclusion of small molecule chemicals. No doubt this was in part because chemicals are both more numerous and their functions cannot be easily inferred through evolutionary analysis. Furthermore, data from the chemical world was (and continues to be) somewhat less electronically and openly accessible. Despite these challenges, some notable efforts have been made to create ontologies for chemistry or chemicals. For instance, ChemOnt (5) and OntoChem (6) are two well-known ontologies developed for describing chemical structures and their relationships. However, these are limited to describing chemical structures in a hierarchical manner—not chemical functions, roles or origins. ChEBI (7) has developed a structured chemical nomenclature that resembles an ontology, but again, this is mostly limited to describing chemical structures. Additionally, the National Library of Medicine has developed MeSH (Medical Subject Headings) terms. MeSH consists of a controlled vocabulary dictionary/thesaurus built around a modest hierarchy that is used for indexing articles for PubMed (8). MeSH covers thousands of structured descriptions for many chemicals. Yet another ontology is the Drug Ontology, or DrOn (9), an ontology developed for drugs. However, drugs represent a very small and specialized class of chemicals and DrOn is primarily focused on describing drug ingredients and treatments, not drug functions or mechanisms.

As far as we are aware, no comprehensive ontology exists to describe chemical functions or chemical origins, certainly not at the level to which GO describes protein/gene functions. Therefore, in an effort to overcome this gap, we developed ChemFOnt: the Chemical Functional Ontology. This represents an effort that has spanned more than five years and has required the work of more than a dozen programmers, curators and database specialists. ChemFOnt (Version 1.0) was created to help unify the description of chemicals and chemical attributes in an effort to create a more comprehensive, computational model of both chemical and biochemical systems. ChemFOnt is intended to bring the same rigor, standardization and formal hierarchical structure to chemistry and biological chemistry as the gene ontology (GO) has brought to molecular biology. In the following pages, we will describe (a) the hierarchical structure of ChemFOnt and its rationale; (b) how ChemFOnt was constructed; (c) the design and implementation of the ChemFOnt resource; (d) ChemFOnt use in other databases and (e) future developments. It is important to emphasize that this is only the first release of ChemFOnt and that many future updates, improvements and expansions are expected over the coming years.

## ChemFOnt STRUCTURE AND RATIONALE

ChemFOnt was designed to follow the same concepts and designs as most well-established hierarchical or tree-based ontologies. As with any ontology, there is a top layer that attempts to encapsulate or summarize one or more broad aspects for the subject area. Each major functional ‘aspect’, functional super-category or parent node in this top layer is then further divided into two or three further child nodes, which are then divided into even more child nodes to form a knowledge tree or a knowledge graph. In ChemFOnt, up to seven layers of parent-child nodes can exist for any of the four major aspect nodes before it reaches its terminal or leaf node. Each functional super-category, category or lower-level child node within ChemFOnt is connected logically to the other by a relationship (or an edge) through a hierarchical taxonomic relationship, a hierarchical structural relationship, a hierarchical anatomical relationship, a hierarchical biochemical/pathway relationship or a hierarchical disease/bioactivity relationship. Most of these other relationships or hierarchies are derived from pre-existing ontologies or pre-existing taxonomies (10–12).

In analogy to GO, ChemFOnt's top hierarchical layer consists of four broad ‘aspects’ or functional super-categories that define a given chemical's effects and origins: (a) *Physiological Effect*; (b) *Disposition*; (c) *Process* and (d) *Role*. These four aspects were chosen based on their ability to help describe chemicals and their relationships to other entities for applications in environmental chemistry, food chemistry, medicinal chemistry, biological chemistry, industrial chemistry, toxicology and pharmacology. These functional super-categories were also designed to fit with a variety of ‘omics’ applications including exposomics, metabolomics, foodomics and lipidomics. As all these fields are constantly evolving and even brand new ‘omics’ fields are appearing, it is expected that the breadth, scope and definitions of these four super-category nodes or their immediate child nodes, will have to change and evolve over time.

The *Physiological Effect* of a chemical is defined as: ‘the measured or observed physiological effect (adverse or positive) on an organism resulting from its exposure to a chemical’. The *Physiological Effect* super-category is intended to provide a clear relationship between a nano-scale chemical and its macro-scale effects, including its organoleptic effects or its clinical/medical consequences. In most cases this refers to the effects on humans or other mammals, although it may be specific to certain types of insects (pesticides), plants (herbicides) or fish and other aquatic species. The relationship terms that should be typically applied to *Physiological Effect* are: ‘Chemical X has a…’ or ‘Chemical X is associated with a…’ or ‘Chemical X causes…’. An example of a *Physiological Effect* in ChemFOnt is the disease diabetes mellitus, which is classified as an endocrine system disorder (the parent node) which, in turn, is classified as a health effect (the grandparent node).

The word *disposition* is normally defined in the dictionary as ‘the way in which something is placed or arranged, especially in relation to other things’. For chemicals we have elaborated on this definition to mean something a bit more specific, namely ‘the manner, the way, or the location in which a chemical can be put into an organism’. For ChemFOnt, we have shortened the definition of *Disposition* to mean: ‘the origin of a chemical, its location within an organism or its route of exposure’. The *Disposition* super-category is intended to provide relationship information or connections between a chemical and its biological or physiological location, its (biological or non-biological) origins or sources and how it can enter an organism. In some cases, the origin may not be known but its location and route of exposure is known. In most cases, the *Disposition* refers to biological locations within humans or routes of entry into humans or other mammals. Likewise, the origin or sources typically refers to its origin with regards to human (or other mammal) consumption, contact or inhalation sources via certain foods, drinks, drugs, aerosols, or skin absorption via the application of cosmetics. The relationship terms that should typically be applied to *Disposition* are: ‘Chemical X comes from…’ or ‘Chemical X is located in the…’. An example of a *Disposition* in ChemFOnt is the term skeletal muscle, which is classified as a muscle tissue (the parent node) which, in turn, is classified a tissue (the grandparent node), which is classified as a biological location (the great grandparent node).

The *Process* for a chemical is defined to mean: ‘the biological or chemical events, or a series thereof, leading to a known function or a known end product’. The *Process* super-category is intended to provide relationship information or connections between a chemical and its more molecular-scale environmental, industrial or biological processes. Most of these processes are associated with chemical reactions, where the chemical is either a substrate or a product or a binding moiety (agonist, antagonist). These reactions may refer to biological, industrial or environmental reactions as well as (biological or environmental) binding/signalling events. A biological or environmental process does not generally refer to a complete pathway but only a module within a given pathway. As most of the processes in ChemFOnt (version 1.0) are biological (for now), most refer to biological pathways or biological reactions. The relationship terms that should be applied to *Process* are: ‘Chemical X is involved in…’ or ‘Chemical X is associated with…’. An example of a *Process* in ChemFOnt is the term histidine metabolism, which is classified as a metabolic pathway (the parent node) which, in turn, is classified a biochemical pathway (the grandparent node), which is classified as a biological process (the great grandparent node), which is further classified as a naturally occurring process.

The *Role* of a chemical is defined as ‘the purpose or function of a chemical, either naturally or as intended by humans’. The *Role* super-category is intended to provide relationship information or connections between a chemical and its adverse and normal biological roles, its environmental roles and its known industrial or commercial applications. While *Role* and *Process* may seem similar, it is important to remember that in ChemFOnt, *Role* is a noun while *Process* is a verb. The relationship terms that should typically be applied to *Role* are: ‘Chemical X plays a role as a…’ or ‘Chemical X is used as a…’. An example of a *Role* in ChemFOnt is the role for 1-methylhistidine in kidney disease, where it is classified as a diagnostic biomarker (parent node), which is classified as a biomarker (grandparent node) which has a role in industry (great grandparent node) as an industrial application (the root node).

Each of the four major super-categories of chemical function is ChemFOnt are divided into two, three or even four child nodes, for a total of 12 major functional categories. For example, *Physiological Effect* is split into two child nodes: *Health Effects* and *Organoleptic Effects*. Likewise, *Disposition* is split into three child nodes: *Sources*, *Biological Locations*, and *Routes of Exposure*. Similarly, *Process* is split into three child nodes: *Environmental Processes, Biological Processes*, (which can also be called Natural Processes) and *Industrial Processes*. *Role* is split into four child nodes: *Adverse Biological Roles, Normal Biological Roles, Environmental Roles* and *Industrial Applications*. These 12 major functional categories are subdivided into another 399 functional subcategories which are further divided into thousands of other branches or leaf nodes for a maximum depth of up to seven layers. In particular, *Physiological Effect* has 3637 defined categories; *Disposition* has 4816 defined categories; *Process* has 161 098 defined categories; and *Role* has 3751 defined categories. In total, ChemFOnt has 173 705 fully defined and fully connected functional categories, which are all placed into a logically consistent hierarchy.

It is important to note that these lower-level functional categories have also been subject to constant adjustment and modification as ChemFOnt has evolved and grown over the past five years. Initially, ChemFOnt was designed to work exclusively for environmental chemistry applications. As it evolved to meet food chemistry, metabolomics and lipidomics applications, new functional categories had to be introduced or modified in ChemFOnt. As ChemFOnt expanded to support microbial and metagenomics, similar changes had to be made. This constant, iterative improvement is essential for the growth of any ontology and will undoubtedly continue as ChemFOnt evolves over the coming years The complete hierarchical structure for ChemFOnt (Version 1.0) is available as an HTML file in the Download section on ChemFOnt's menu bar.

Each of the functional categories in ChemFOnt terminates to a disease, condition, pathway, role, source, industrial/commercial process, routes of exposure, biological or physiological structure, taxonomic species, protein/enzyme or reaction. These terminal or leaf nodes also contain a fully defined term (for a total of 173,305 definitions). Similarly, every chemical in ChemFOnt is also defined (for a total of 341 627 definitions) and every chemical structure or structure class is also defined via ClassyFire (5) (for a total of 4825 definitions). The entire ontological hierarchy for ChemFOnt currently comprises a total of 515 332 fully defined terms, which are linked to ∼10.3 million chemical/functional relationships. Furthermore, all terminal leaf nodes in the ChemFOnt hierarchy contain a hyperlinked fact or statement supported by one or more citeable references. In other words, every fact or factoid in ChemFOnt has a traceable provenance.

## BUILDING AND ANNOTATING ChemFOnt

Unlike GO, which was annotated through many years of manual curation by a global community of thousands of volunteer annotators and curators, ChemFOnt was annotated primarily through the activities of past and present members of a single laboratory (the Wishart laboratory). While it should be noted that all 12 of the functional category definitions in ChemFOnt, along with the hierarchical structure were manually written, adjusted, formatted and curated, most of the other data in ChemFOnt was assembled and annotated using automated or semi-automated processes. In particular, ChemFOnt curators made extensive use of automated data extraction and data harvesting techniques from local electronic databases that had been manually assembled and curated. The automated annotation process used for ChemFOnt was particularly important for accommodating its constantly evolving and constantly expanding hierarchical and categorical structure (see above).

Automated or semi-automated annotations were primarily performed by mining the manually curated and manually maintained chemical and biological databases already housed and maintained in the Wishart laboratory. For instance, all 341 627 chemical definitions were automatically obtained from the descriptions/definitions available from Human Metabolome Database (HMDB) (13), Natural Products Magnetic Resonance Database (NP-MRD) (14), DrugBank (15) and FooDB (16). Similarly, all 19 391 protein definitions were harvested from the descriptions/definitions available from UniProt (17). Food sources, food taxonomies, food biomarker data, food definitions and many organoleptic effects were extracted from FooDB (16) and Food-Biomarker Ontology (FOBI) (18). Most health effects (and statements corresponding to abnormal biofluid levels), biological locations and industrial/commercial roles were extracted from HMDB (13), DrugBank (15), Toxin and Toxin Target Database (T3DB) (19) and MarkerDB (20). Chemo-taxonomic information for most chemicals in ChemFOnt was harvested from HMDB (13), PathBank (21), Microbial Metabolome database (MiMeDB) [https://mimedb.org], NP-MRD (14) and FooDB (16). Pathway and chemical reaction data was pulled from Small Molecule Pathway Database (SMPDB) (22), HMDB (13) and DrugBank (15). Similarly pharmacological, toxicological and industrial application data was harvested from DrugBank (15), T3DB (19) and MarkerDB (20). Cell, tissue and other anatomical names and locations were constructed from GO (1) and the NCI Thesaurus (11). Disease names, disease definitions and disease hierarchy information were pulled from MarkerDB (20) and Systematized Nomenclature of Medicine—Clinical Terms (SNOMED-CT) (23).

Because nearly all the databases mentioned above were constructed using a mySQL framework, a variety of SQL scripts or queries were written and used to extract and reformat the relevant data from the labeled data fields in each of the above-mentioned databases. For those data sources that were only available as downloadable ontologies or downloadable files (available as OWL or CSV- comma-separated values formatted files), similar extraction queries or tools were written using Python. These same SQL or related scripts were also used to extract the relevant references or citations to provide the required provenance for each ontological fact in ChemFOnt's leaf/terminal nodes. Additionally, a variety of Python scripts were written to perform data mining of the detailed text descriptions for all the chemicals and all the pathways housed in the above-mentioned databases. This was done to map and extract specific functional terms (such as specific health effects, food sources, microbial sources, relevant proteins, functional roles, processes, etc.) and their associated references so that they could be added to the ChemFOnt annotations. Spot checks and cross checks were constantly performed by the ChemFOnt team to ensure that the extracted data matched exactly to the data from the source and that it was categorized correctly.

In addition to these automated or semi-automated ‘bottom up’ annotations (via electronic data harvesting from our local databases), a manual, top-down annotation was performed by several ChemFOnt curators. This manually intensive effort was done to ensure that large collections of chemically or functionally similar, but lightly annotated compounds (esp. lipids, plant/microbial natural products, food additives and environmental contaminants) would have an appropriate level of ChemFOnt annotation. As with the automatically harvested annotations, all the manually assembled ChemFOnt annotations were spot checked for completeness and consistency with their source materials by a separate team of curators and annotators to ensure correctness and completeness. Table 1 provides more detailed statistics regarding the number of terms, functional categories, references, and identified relationships in ChemFOnt. As can be seen from this table, ChemFOnt is already comparable in size and scope to GO.

**Table 1.** Number of terms, functional categories, references, and identified relationships in ChemFOnt

**Descriptions**	Number
Defined terms	515 332
Total words (terms and definitions)	∼85 000 000
Major categories	399
Links to metabolites	10 298 339
References supporting relationships	11 191
Compounds (Chemicals)	341 627
Compound classifications	4825
Proteins	23 819
Functional terms	173 705
Pathways	111 429
Biological roles	642
Biological locations	746
Industrial applications	1320
Metabolite–protein relationships	3 974 409
Protein–pathways relationships	886 517
Metabolite–cellular location relationships	387 202
Metabolite–tissue location relationships	113 589
Metabolite–gene ontology (GO) terms relationships	4 148 816
Metabolite–pathway relationships	12 371 764

The complete set of ChemFOnt annotations along with their corresponding ChemFOnt functional categories, their supporting or clarifying statements and their provenance information were uploaded into a specially constructed MySQL database. This database served as both the back end and the underlying data framework to construct the web-enabled ChemFOnt database (described below) as well as the downloadable OWL files, the HTML hierarchy file and the corresponding downloadable CSV files that are all available through ChemFOnt's online download tab.

## ChemFOnt WEB INTERFACE, DOWNLOADS AND UPDATES

**Figure 1.**
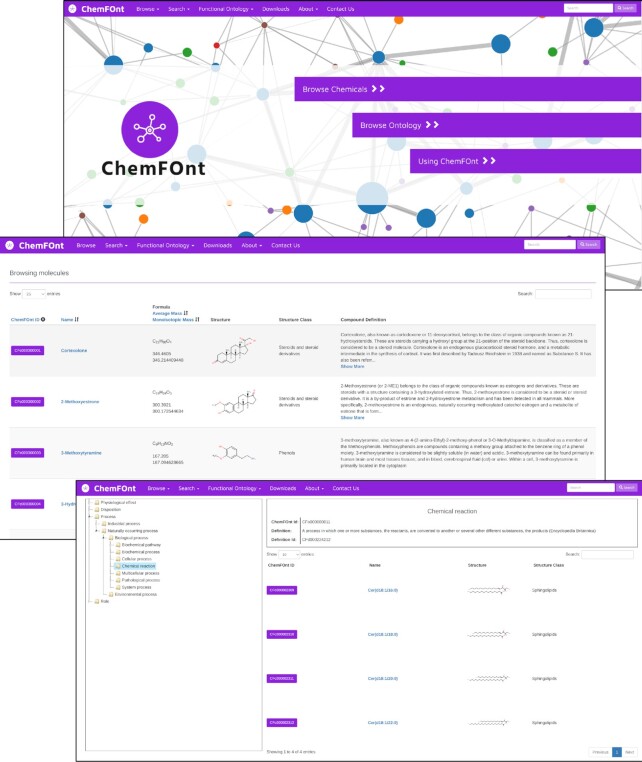
Screenshot montage of the Chemical Functional Ontology Database (ChemFOntDB) showing the Homepage at the top and the Molecule Table (middle) describing each chemical using six different columns of information and the ChemFOnt Ontology Browser at the bottom.

The web-enabled version of ChemFOnt adheres to many of the same interface features and layout as many of our databases such as the HMDB (13), *Escherichia coli* Metabolome Database (ECMDB) (24) and Yeast Metabolome Database (YMDB) (25). A screenshot of the ChemFOnt homepage is shown in Figure 1. As seen in the top image the website's purple navigation bar (located at the top) consists of six menu options: **Browse**, **Search**, **Ontology**, **Downloads**, **About** and **Contact Us**. On the right side is a general text search box where users may enter text to search the entire ChemFOnt database. Below the navigation bar is a set of three purple hyperlink bars that allow users to instantly access key parts of ChemFOnt, namely: (i) *Browse Chemical Data*, (ii) *Browse Ontology Data* and (iii) *Using ChemFOnt*. Clicking any one of these hyperlink bars will take users to the (a) ChemFOnt Chemical Data Browser, the (b) ChemFOnt Ontology Browser and (c) ChemFOnt's tutorial on how to use ChemFOnt.

Clicking the Browse Chemical Data hyperlink on the main page or the Browse option on the purple navigation bar will immediately generate a browsable table called a Molecule Table. The Molecule Table displays six columns (Figure 1) describing each chemical in ChemFOnt. This includes the ChemFOnt ID (or ChemFOnt compound identifier), the Name, the Molecular Formula, the Mass (Average and Isotopic), a thumbnail image of the Structure, the Structure Class, and the Compound's Definition. Several of the columns are sortable (ChemFOnt ID, Mass and Name) and can be sorted by clicking on the arrows beside each column name. The Structure Class refers to the chemical class to which the compound belongs using the ClassyFire taxonomy (5). Users may browse through the table by scrolling down with their mouse or trackpad or by jumping from page to page with the numbered pagination buttons located on the top left or bottom left of the Molecule Table page. By default, 25 compounds are displayed in each Molecule Table.

Clicking on the purple CompoundCard button (in the first column) for any compound listed in the Molecule Table generates a full view of the CompoundCard. Each ChemFOnt CompoundCard contains seven data fields. These include: (i) ChemFOnt Record Information; (ii) Molecule Identification; (iii) Chemical Taxonomy; (iv) Functional Ontology; (v) Physical Properties; (vi) External Links and (vii) References. Each of the named data fields are highlighted by a purple background with some of them (Molecule Identification, Functional Ontology and Record Identification) always opened by default. Each of the data fields in a ChemFOnt CompoundCard may be expanded or contracted by clicking the down arrow on the right side of each of the purple bars with the corresponding data field name. Users may return to the Molecule Table by clicking on the back arrow of their browser or the Browse button at the top of the Navigation bar.

The ChemFOnt Record Information field contains the ChemFOnt version number, the date it was created and last updated and a unique, nine-digit ChemFOnt chemical identification number. The Molecule Identification field contains data on the compound's common name, its definition (often including a detailed description), an image of the structure, various names or synonyms, the chemical formula and molecular weight (average and monoisotopic), a Chemical Abstracts Service (CAS) number as well as the SMILES (Simplified Molecular Input Line Entry System) and InChI (International Chemical Identifier) identifiers. The Chemical Taxonomy data field displays the ClassyFire (5) taxonomy. This data field provides a brief description, the major chemical (structural) classes to which it belongs, information about its chemical substituents, the main molecular framework that defines the structure as well as an external database descriptor. The most important data field is the Functional Ontology field. This data field displays the hierarchically structured ChemFOnt ontology with each of the four main functional aspects or super-categories (*Physiological Effect, Disposition, Process and Role*) for the given chemical listed on the left-most column and the corresponding child categories listed in a progressive, tab-delimited format on the right. Hovering over any of the terms with a mouse or track-pad pointer will cause the term's definition to appear in a black ‘definition box’. The terminal category or terminal node in a given hierarchy is always colored dark blue and if users click on the hyperlinked word, it directs users to the corresponding Wikipedia page (if available) or a corresponding web page in MarkerDB, or PathBank providing more information about the subject. Beside each terminal category term is a reference or citation that explains why/how the compound is associated with that term and provides one or more hyperlinked references (to a PubMed ID, a DOI, or an online database entry). The remaining data fields, Physical Properties, External Links and References are mostly self-explanatory and will not be discussed further.

If users click on the ChemFOnt Ontology Browser, a browsable, interactive tree view of ChemFOnt's full ontology is generated. Users can browse, select and change the different ontology levels by clicking each level. The top right corner of the Ontology Browser displays a unique nine-digit ChemFOnt ontology identification number that is assigned to each ontology term in ChemFOnt. The Ontology Browser also displays information on all ChemFOnt definitions and each ChemFOnt definition is also assigned a unique nine-digit ChemFOnt definition number as shown in the bottom right corner. The Ontology Browser also has a search box where users can enter text data or search for any of the three different types ChemFOnt identification numbers.

ChemFOnt also provides several **Search** options. On the upper right-hand corner of the main page (Figure 1), a text search box is available that allows users to search ChemFOnt by text. After typing the desired text in the box, users must press the purple ‘Search’ button to activate the text search. As with the most modern text search utilities, an auto-suggest feature is provided to help facilitate the search and perform spelling corrections. ChemFOnt offers three other search options located on the top navigation bar via the **Search** menu tab. These options include ChemQuery Structure Search (for chemical structure searches), a standard Text Query Search, and an Advanced Search. The ChemQuery Structure Search utility uses the MarvinView Applet from ChemAxon, which allows users to interactively draw structures (or paste InChI or SMILES strings) into an interactive drawing pallet and to search for similar chemical structures using the Tanimoto similarity index. The other text query searches are simple to use and mostly self-explanatory.

ChemFOnt also has an **Ontology** tab, a **Download** tab, an **About** tab and a **Contact Us** tab. The **Ontology** tab provides a pulldown menu where users may select an overview of ChemFOnt (similar to this manuscript), a guide to the ChemFOnt subsets and instructions for contributing to ChemFOnt. The **Download** tab allows users to access most of the ChemFOnt data including the SQL (PostgresSQL) version of ChemFOnt, the OWL version of ChemFOnt, the chemical structure files (in SDF), the list of ChemFOnt functional categories and their definitions (in CSV), the HTML file describing the ChemFOnt hierarchical structure along with information on their size, file format type and a brief description of their contents. The **About** tab has a submenu that provides an overview on the ChemFOnt project, ChemFOnt statistics, citation information/help- a brief tutorial on how to use ChemFOnt, licensing details and information on its FAIR compliance.

Improvements and updates to ChemFOnt's content and structure have been done and will be done on a continuous basis. Throughout its development, ChemFOnt curation and programming team members met weekly to design the ontology architecture, write definitions, coordinate annotations, track team member activities, perform quality assurance checks and ensure that tasks were completed in a timely manner. This same process is expected to continue for the foreseeable future. Minor corrections or small additions to any given ChemFOnt entry or its layout are typically done without a formal update announcement. However, all changes are tracked internally and external users can see from the last update date when any changes were made. As this is only version 1.0 of ChemFOnt, all ChemFOnt entries are annotated with August 2022 as the last update date. Large-scale updates and improvements to ChemFOnt in the future will be given database version numbers (2.0, 3.0, etc.) and suitable database update timestamps.

The ChemFOnt website uses standardized frameworks and caching systems to make the website more user friendly and responsive. As with other databases developed by our group, ChemFOnt uses Redis-based caching that makes the loading of data, structures and images very fast. To facilitate rapid prototyping and development, the entire ChemFOnt database was built upon an MVC (Model-View-Controller) framework called Ruby on Rails (version 7.0.3). In this MVC framework, models respond and interact with the data by connecting to the database, views create the interface to show and interact with the data, and controllers connect the user to the views. Such a framework allowed our database developers to easily create code for much of ChemFOnt. This framework is particularly robust and code can be reused in different functions or changed easily to accommodate future plans or abrupt changes in design. In particular, this allowed our development team to liberally borrow code and functions from other databases developed in our lab (13–16,18–22). ChemFOnt is housed in the Cloud (hosted by Digital Ocean) on a Quad virtual processor 1.8 GHz system with 8GB of RAM and 410GB of hard disk space. The entire ChemFOnt database occupies 2.1 Gbytes.

## ChemFOnt IN OTHER DATABASES AND SOFTWARE TOOLS

**Figure 2.**
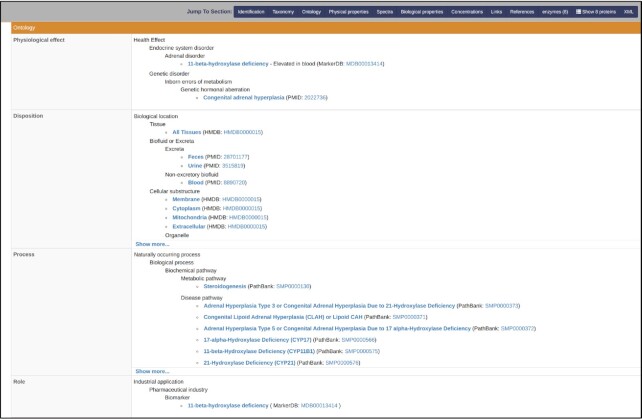
Screenshot showing how easily ChemFOnt can be integrated into an existing chemical database using the human metabolome database (HMDB) as an example.

An ontology is only useful if it is widely adopted and used by other software tools or database resources. Indeed, many ontologies, as with many data exchange standards, have essentially disappeared because of little community uptake or poor community buy-in (26). Our decision to develop ChemFOnt was partly driven by a growing community need to establish higher quality and more consistent functional descriptors for chemicals with the intention to make both chemical and biochemical information ‘more computable’ or more machine readable. In addition, the development of ChemFOnt was also driven out of our own need to standardize terms and terminology within our growing collection of curated chemical databases (such as HMDB, NP-MRD, DrugBank, FooDB, MiMeDB, PathBank and others). As a result, ChemFOnt has already been fully integrated into the latest release of HMDB (13). By the end of 2022, ChemFOnt will also be integrated into NP-MRD (14), MiMeDB [https://mimedb.org] and FooDB (16) as well as other databases in our collection. Discussions are underway with other database providers to incorporate ChemFOnt into their online chemical databases. An example of how ChemFOnt can be easily integrated into an existing chemical database (HMDB) is shown in Figure 2. As currently structured in HMDB, the ChemFOnt ontology is only searchable via the Advanced Text Search function. Additional data filters and more advanced search or visualization tools to more fully exploit the information in ChemFOnt will be added to HMDB (and other database) over the coming year.

## FUTURE DEVELOPMENTS

This is the first of what we hope will be many releases and updates of ChemFOnt. Clearly with only ∼342 000 chemicals in the current release of ChemFOnt, we are not covering all of the biologically relevant chemical space. Based on data regarding known natural products (27), known chemical contaminants and industrially produced chemicals (28,29) and what we know is currently in ChemFOnt, we estimate are that there are ∼1 000 000 chemicals that may be routinely produced or encountered by living organisms. This is obviously much less than the number of compounds in PubChem (30) or ChemSpider (31) but considerably more than what is currently in HMDB or NP-MRD. The reason for the discrepancy is that the vast majority (>99.9%) of the compounds reported in PubChem, ChemSpider and other large chemical repositories are synthetic compounds, prepared in miniscule quantities for research-only or screening-only purposes. While these synthetic compounds may have interesting biological or physiological consequences, they are unlikely to have been released in the natural world or to be encountered by anyone except the individual (or robot) that synthesized the compound.

Consequently, our focus over the coming years will be to expand ChemFOnt to cover all of the ∼1 000 000 compounds that are ‘encounterable’ in the natural world. In addition to expanding ChemFOnt's coverage, we also plan to enrich the data within ChemFOnt by exploiting more sophisticated natural language processing (NLP) tools to mine existing literature. We have developed and experimented with some of these tools (32) and intend to make more use of deep learning techniques to extend their capabilities (33). The use of NLP should reduce the burden of manual curation while at the same time extending the breadth, quantity and quality of citable information about encounterable chemicals and their functional properties.

Over the next two to three years, we intend to expand the use of ChemFOnt in our own databases and in our own software tools. This will include developing specific, open-access tools to better filter, search and display ChemFOnt information in our databases, as well as APIs to help external users mine or extract data from ChemFOnt. Similarly, efforts will also be made to ensure that ChemFOnt is more widely used in other popular, online chemical databases such as PubChem (30), ChemSpider (31), LIPID MAPS (34), the CompTox Chemicals Dashboard (35), COCONUT (27) and ChEBI (7). A major effort will also be undertaken to integrate ChemFOnt into a variety of multi-omics data analysis tools such as MetaboAnalyst (36), Galaxy for Metabolomics (37) and various metabolite set enrichment tools (38,39).

Overall, we believe ChemFOnt represents an important first step in the path to make functional information about the chemicals in our world more accessible, more machine readable and more ‘computable’. We also believe that ChemFOnt will be able to help unify the description of chemicals and chemical attributes so that a more comprehensive, computational model of biochemical systems can be created. This should enable much better integration of functional chemical information into metabolomics, proteomics, genomics and metagenomics data analysis workflows. Ultimately, ChemFOnt is intended to bring the same rigor, standardization and formal hierarchical structure to chemistry and biological chemistry as the gene ontology (GO) has brought to molecular biology.

## DATA AVAILABILITY

ChemFOnt is available as both a freely accessible, web-enabled database and a downloadable Web Ontology Language (OWL) file.
